# Transition patterns of weight status: A cohort study of Chinese school-age children

**DOI:** 10.3389/fpubh.2022.942307

**Published:** 2022-11-07

**Authors:** Xin Hu, Linglin Tan, Zhaoxin Wang, Jing Zhang

**Affiliations:** ^1^School of Public Health, Shanghai Jiao Tong University, School of Medicine, Shanghai, China; ^2^Department of Occupational and Environmental Health Sciences, Peking University School of Public Health, Beijing, China; ^3^Qibao Community Health Service Center of Minhang District, Shanghai, China; ^4^The First Affiliated Hospital, Hainan Medical University, Haikou, China; ^5^School of Management, Hainan Medical University, Haikou, China

**Keywords:** childhood obesity, weight status, cohort study, multistate model, transition probability

## Abstract

**Background:**

Childhood overweight and obesity are increasing public concerns. However, little is known about the transition patterns of childhood weight status, especially in developing countries. In this study, we aimed to evaluate patterns of change in weight status and the risk factors among Chinese school-age children.

**Methods:**

This retrospective cohort study included 2,334 children aged 6 years with complete 5-year (2012–2017) physical examination data in Minhang District, Shanghai. A time-homogeneous three-state Markov model was fit to the longitudinal data with dynamic outcomes (normal weight, overweight, and obesity).

**Results:**

According to the Markov model, 42.3% of school-age children who were initially overweight transitioned to another weight status within 1 year, with 24.8% (95% confidence interval [CI]: 23.1, 27.0) transitioning to normal weight and 17.5% (95% CI: 15.9, 19.3) becoming obese. In contrast, children who were initially normal weight (92.9% [95% CI: 92.3, 93.5]) or obese (83.1% [95% CI: 81.1, 84.8]) tended to maintain their initial weight status. Male sex, semi-urban area, absence of late adiposity rebound, lower annual height increments, higher annual weight increments, and higher initial body mass index were significantly associated with a higher risk of developing or maintaining overweight and obesity (*p* < 0.05).

**Conclusions:**

The weight status of Chinese school-age children is more likely to change among those who are initially overweight than in those who are initially obese. Interventions to promote healthy weight status may be more effective if key groups are targeted, such as overweight and pre-school-age children.

## Introduction

Childhood overweight and obesity are increasing concerns, with rates now experiencing a dramatic rise. From 1975 to 2016, the prevalence of overweight or obesity among children and adolescents aged 5–19 years increased more than 4-fold globally from 4 to 18% (1). China has the largest number of obese children (2), and the prevalence of obesity among Chinese school-age children (age 7–18 years) increased from 0.1% in 1985 to 7.3% in 2014 (3). Moreover, it has been predicted that by 2030, the prevalence of overweight and obesity could reach 31.8% among school-age children and adolescents in China (4). Childhood overweight and obesity are important determinants of adult health. Studies have shown that 55% of childhood obesity persists into adolescence, 80% of obese adolescents will still be obese in adulthood (5), and obesity is significantly associated with an increased risk of coronary heart disease, metabolic syndrome, and psychological disorders (6, 7). Considering that ~22% of the national medical expenditure in China might be attributable to obesity in 2030 (4), in-depth understanding of childhood weight status is of great importance to public health and social economics.

Most current studies are focused on the prevalence of childhood overweight and obesity (8–11). Although these prevalence estimates provide information on epidemiology and disease burden, little is known about the dynamic transitions of weight status. As children grow, their weight status might change depending on their physical maturation, psychosocial factors, dietary habits, and physical activity (3). Taking into account that overweight and obesity are recurrent and modifiable, exploring the transition patterns of weight status could be used to determine the transition probabilities across age groups, the expected time that children remain within each weight status, and the influence of baseline characteristics such as sex and school area. Policy makers aiming to reduce the burden of childhood obesity may benefit from such studies, which can help in identifying the optimal intervention groups and effect modifiers.

To date, only a few studies, conducted mostly in developed countries, have used a multi-state Markov model to investigate childhood weight status. However, the findings of these studies have varied. In 2014, Lohrmann et al. (12) reported that both overweight and obese students in Pennsylvania (United States) tended to transition to another weight status, with only 48.14% of overweight and 24.38% of obese children maintaining their initial weight status over a 2-year period. Another study in Colorado (United States) found similar results in that children who were overweight at age 3 years were expected to remain in overweight status for only 5.1 of the following 13 years, with more than half of the next 13 years spent in other weight statuses (13). In contrast, a Finnish study in 2020 found that overweight and obese statuses tended to be maintained, with 76.3% of initially overweight girls remaining overweight after 1 year, a higher proportion than the 63.4% for normal weight and 67.7% for obesity (14). Currently, the evidence is limited from developing countries. To our knowledge, only one study conducted in northern China investigated the transitions of childhood weight status; however, that study was characterized by a small sample size (*n* = 928) and was conducted from 2004 to 2009 (15). There is evidence indicating a rapid increase in the obesity epidemic among Chinese children (11). Therefore, further large studies in developing countries with more recent data are needed.

In this study, we used data of a 5-year fixed cohort (*n* = 2,334) and applied a continuous-time multistate Markov model stratified by sex to assess the transition patterns of childhood weight status and identify risk factors among Chinese school-age children. We put forth the following hypotheses: (1) the probability of transitioning or maintaining weight status among school-age children differs by weight status; (2) the transition patterns of school-age children are influenced by characteristics such as sex, school area, and early growth trajectories (adiposity rebound, AR).

## Materials and methods

### Participants

This retrospective cohort study included 2,334 consecutively enrolled children who had participated in the annual physical examination program for primary and secondary school students in Minhang District, Shanghai between 2012 and 2017. Considering the dissimilarities in growth, development, and weight status transitions among different age groups of children (16, 17), we selected the youngest group of school-age children, those who were 6 years old in 2012, to better explore the transition patterns of weight status with age and to prevent mixing of these patterns in different age groups. Children who had no diseases associated with pathological obesity were eligible for inclusion. We excluded those who had any cardiac, hepatic, renal, or other severe acute/chronic diseases and those with missing data from the physical examination.

We extracted secondary data from the electronic records established for the physical examination program managed by the local center for disease control and prevention. During the physical examination, children's height and weight were measured and personal information was recorded by professionally trained community physicians or advanced practice registered nurses from community health centers. Weight and height were measured with the children wearing no shoes and light clothing. Weight was measured to the nearest 0.01 kg using a Tanita BC-420 digital scale, and height was measured to the nearest 0.1 cm with an SG-210 stadiometer. Each student was assigned a unique identification number matching the examination record and personal information, which included sex, grade, school, and birthdate. Details of this program have been reported previously (16).

### Evaluation of variables

Body mass index (BMI) was calculated as weight (in kilograms) divided by the square of height (in meters). We used the BMI reference norms recommended by the Group of China Obesity Task Force (18) to categorize weight status as normal weight, overweight, or obesity in children aged 7–11 years. This is a widely applied standard for Chinese school-age children and is consistent with the ethnic characteristics of East Asian populations. Given that the abovementioned standard was developed for children aged 7–18 years and a classification standard for 6-year-old children is lacking, we used the classification criteria of the World Health Organization (19) to classify the weight status of 6-year-old children (https://www.who.int/tools/growth-reference-data-for-5–19years/indicators/bmi-for-age). Those two standards have been frequently used in studies investigating the weight status of Chinese children of different age groups (3, 20).

Adiposity rebound (AR) refers to an increase in a child's BMI curve after having reached a nadir at approximately 4–8 years of age. Early AR is associated with an increased risk of adulthood obesity (21). We applied a mixed-effects linear model to fit the BMI curves of each child and defined the timing of AR as the age at which the child had the lowest BMI, as previously reported (22). Children whose AR occurred at or after age 7 years were categorized as having late AR. The remaining children were classified as not having late AR; this group included two children whose derivative BMI curves lacked a zero value and 1,016 children whose AR was estimated to be earlier than age 7 years. Participants' ages were computed according to the date of birth and date of physical examination. Urban and semi-urban areas were defined according to the urbanization level of the street or town where each school was situated. Annual height increment (AHI) and annual weight increment (AWI) were defined as the average increase in height and weight from 1 year to the next, thus these values were unavailable for the last year. The initial BMI was defined as the BMI at the time each child entered the cohort (at age 6 years).

### Statistical analysis

We used the time-homogeneous multistate Markov model as revised by Jackson (23) to fit the transition pattern of childhood weight status. Unlike direct calculation of the incidence and remission rate, logistic regression models, and Cox proportional hazard models, the multistate Markov model can provide insight into the dynamic nature of multiple weight status and has the following strengths. First, the multistate Markov model can calculate the instantaneous risk of transition between any two states. Second, this model allows for unequal time intervals between two consecutive observations. Third, this model can take into account changes in covariates over time and evaluate their effects on multiple transitions. Lastly, the multistate Markov model addresses bias associated with “interval censoring,” that is, unobserved intervals between two observations. Previous relevant studies have applied this model and have confirmed that the transition process across weight statuses is in accordance with the Markov assumption (24, 25).

We applied a three-state (normal weight, overweight, and obesity) model stratified by sex, and set the condition that instantaneous transition can only occur between adjacent statuses, that is, changes between normal weight and obesity must pass through overweight status. To minimize the effects of random fluctuations resulting from varying starting points, we defined the time scale *t* as the time since the first measurement. At first, we fit the null model with no covariates included and computed the transition intensities, mean sojourn time, transition probabilities, and total length of time spent in each state. Next, we input sex, area, late AR, AHI, AWI, and initial BMI values into the null model, respectively, and tested goodness-of-fit of the new model by applying the likelihood ratio test and Akaike information criteria (AIC). Considering the collinearity among variables (AWI, AHI, late AR, and initial BMI), we did not develop a model with multiple covariates. Hazard ratios (*HR*s) and their 95% confidence intervals (CI) were then calculated and forest plots were drawn. Finally, we compared the predicted prevalence of the three states in the fifth year (*t* = 5.0) with the observed prevalence in 2017 to validate the estimated probabilities. Validity was confirmed if the estimated prevalence was within the 95% CI of the observed values or if the differences were <1% of the predicted prevalence.

Depending on the type and distribution of variables, we used the χ^2^ test, *t*-test, or Mann–Whitney test to evaluate group differences. *p* < 0.05 was considered to denote statistical significance. We used Excel 2019 for data entry, the “msm” package in R version 4.0.3 (R Foundation for Statistical Computing, Vienna, Austria) for statistical analysis, and GraphPad Prism version 8.0.0 (GraphPad Software, San Diego, CA, USA) to construct the forest plots.

## Results

This cohort included 2,234 children during the study period. Table 1 presents participants' baseline characteristics in 2012. Among the 2,334 children, 49.7% were girls (sex ratio of boys to girls was 1.01), 78.9% attended schools in urban areas, and 37.9% of children had late AR. The median participant age was 6.8 years and the duration of follow-up was 5 years. At baseline, 18.1% of children were overweight and 10.8% were obese, with median BMI equal to 15.9 kg/m^2^. Overweight and obesity were significantly more prevalent among boys than among girls (*p* < 0.001). Among a total of 11,670 observations (*n* = 2,334 with five follow-ups), there were 549 transitions from normal weight to higher weight statuses (512 to overweight and 37 to obesity) and 279 transitions from obesity to other weight statuses (25 to normal weight and 254 to overweight). Additionally, 457 transitions from overweight to normal weight and 336 from overweight to obesity occurred.

**Table 1:** Characteristics of included children in 2012 (*n* = 2,334).

**Characteristics**	**All children**	**Girls**	**Boys**	***p* for sex difference**
**Sex**, ***n*** **(%)**	2334 (100)	1160 (49.7)	1174 (50.3)	
**Baseline weight status**, ***n*** **(%)**				<0.001
Normal weight	1659 (71.1)	918 (79.1)	741 (63.1)	
Overweight	422 (18.1)	175 (15.1)	247 (21.0)	
Obesity	253 (10.8)	67 (5.8)	186 (15.8)	
**Area**, ***n*** **(%)**				0.104
Urban area	1842 (78.9)	932 (80.3)	910 (77.5)	
Semi-urban area	492 (21.1)	228 (19.7)	264 (22.5)	
**Late adiposity rebound**, ***n*** **(%)**				<0.001
With late adiposity rebound	884 (37.9)	498 (42.9)	386 (32.9)	
Without late adiposity rebound	1450 (62.1)	662 (57.1)	788 (67.1)	
Age (year), median (IQR)	6.8 (0.3)	6.8 (0.3)	6.8 (0.2)	0.111
Height (cm), median (IQR)	122.4 (6.7)	122.0 (6.6)	123.0 (6.5)	<0.001
Weight (kg), median (IQR)	23.8 (5.1)	23.1 (4.3)	24.5 (5.6)	<0.001
BMI (kg/m^2^), median (IQR)	15.9 (2.4)	15.6 (2.1)	16.3 (2.7)	<0.001
AHI (6–7 years old, cm), median (IQR)	5.7 (1.8)	5.7 (1.9)	5.8 (1.8)	0.623
AWI (6–7 years old, kg), median (IQR)	2.6 (3.0)	2.4 (2.7)	2.9 (3.1)	<0.001

IQR, interquartile range; BMI, body mass index; AHI, annual height increment; AWI, annual weight increment.

Table 2 shows transition probabilities over 1 and 5 years and the total time spent in each weight status, estimated by the null model. The total length of time was longest for normal weight, averaging 0.962 (95% CI: 0.959, 0.965) years over 1 year and 4.347 (95% CI: 4.298, 4.395) years over 5 years. Among children who were initially categorized as normal weight, 6.3% (95% CI: 5.8, 6.8) and 0.8% (95% CI: 0.7, 0.9) transitioned to overweight and obesity, respectively, within 1 year. Additionally, 2.7% (95% CI: 2.3, 3.1) and 14.2% (95% CI: 12.8, 15.9) of children who were obese at baseline reverted to normal weight or overweight, respectively, over 1 year. As for overweight children, 24.8% (95% CI: 23.1, 27.0) reverted to normal weight and 17.5% (95% CI: 15.9, 19.3) progressed to obesity. For all weight statuses, children were more likely to maintain their initial status than transition to another status during the first year. However, over 5 years, only 20.1% (95% CI: 18.6, 21.8) of children who had been overweight at baseline remained overweight.

**Table 2:** Probabilities (95% confidence intervals) of 1 and 5 years transition between normal weight, overweight, and obesity and the total length of time spent in each weight status.

**Initial weight status**	**Follow-up weight status**			
	**Normal weight**	**Overweight**	**Obesity**	**Total length of time (year)**
**1-year follow-up**			
**All children**				
Normal weight	92.9 (92.3, 93.5)	6.3 (5.8, 6.8)	0.8 (0.7, 0.9)	0.962 (0.959, 0.965)
Overweight	24.8 (23.1, 27.0)	57.7 (55.4, 59.7)	17.5 (15.9, 19.3)	0.035 (0.032, 0.038)
Obesity	2.7 (2.3, 3.1)	14.2 (12.8, 15.9)	83.1 (81.1, 84.8)	0.003 (0.003, 0.003)
**Girls**				
Normal weight	94.6 (93.9, 95.2)	4.7 (4.2, 5.3)	0.7 (0.6, 0.9)	0.971 (0.967, 0.974)
Overweight	27.1 (24.1, 30.2)	54.3 (50.7, 57.5)	18.7 (16.4, 21.4)	0.026 (0.023, 0.030)
Obesity	4.0 (3.2, 4.8)	18.3 (15.6, 21.2)	77.7 (74.2, 81)	0.003 (0.002, 0.003)
**Boys**				
Normal weight	90.9 (89.9, 91.8)	8.1 (7.3, 9.0)	1.0 (0.8, 1.2)	0.951 (0.946, 0.956)
Overweight	23.2 (20.8, 25.9)	60.1 (57.3, 63)	16.7 (14.7, 19.1)	0.045 (0.041, 0.050)
Obesity	2.1 (1.7, 2.5)	12.2 (10.5, 14.1)	85.8 (83.6, 87.7)	0.004 (0.003, 0.004)
**5-year follow-up**				
**All children**				
Normal weight	78.6 (76.8, 80.1)	13.2 (12.2, 14.2)	8.2 (7.4, 9.2)	4.347 (4.298, 4.395)
Overweight	52.3 (49.3, 54.9)	20.1 (18.6, 21.8)	27.6 (25.1, 30.3)	0.469 (0.433, 0.506)
Obesity	26.5 (23.9, 29.2)	22.5 (20.7, 24.4)	51.0 (47.6, 54.8)	0.184 (0.164, 0.207)
**Girls**				
Normal weight	83.6 (81.5, 85.6)	10.1 (8.9, 11.6)	6.3 (5.3, 7.4)	4.499 (4.442, 4.556)
Overweight	57.8 (53.3, 62.3)	18.4 (16.0, 20.9)	23.8 (20.1, 27.4)	0.356 (0.313, 0.398)
Obesity	35.2 (30.6, 40.2)	23.4 (20.7, 26.2)	41.4 (35.6, 46.6)	0.145 (0.122, 0.173)
**Boys**				
Normal weight	72.9 (70.2, 75.2)	16.8 (15.2, 18.5)	10.3 (8.9, 11.8)	4.168 (4.094, 4.248)
Overweight	47.9 (44.0, 51.9)	22.2 (19.9, 24.5)	29.9 (26.3, 33.6)	0.605 (0.546, 0.660)
Obesity	21.5 (18.5, 25.1)	21.8 (19.2, 24.4)	56.7 (51.3, 61.5)	0.227 (0.191, 0.262)

Results were estimated by the null model (with no covariates included).

Table 3 shows the transition intensity matrix and mean sojourn times estimated by the null model. For both boys and girls, the shortest mean sojourn time was for overweight and the longest was for normal weight, suggesting that overweight is relatively unstable whereas normal weight tends to persist. The rate of transitioning from normal weight to overweight was higher for boys (*p* < 0.001). In contrast, the rate of transitioning from overweight or obesity to another weight status was lower for boys (overweight: *p* = 0.006; obesity: *p* < 0.001).

**Table 3:** Transition matrix for a multistate model of transitions between weight status and mean sojourn time for each weight status.

**Initial weight status**	**Follow-up weight status**			**Mean sojourn time (year)**
	**Normal weight**	**Overweight**	**Obesity**	
**All children**				
Normal weight	−0.086 (−0.094, −0.079)*	0.086 (0.0790, 0.094)*	0	11.620 (10.666, 12.659)*
Overweight	0.341 (0.311, 0.374)	−0.598 (−0.641, −0.558)*	0.257 (0.231, 0.285)*	1.673 (1.560, 1.793)*
Obesity	0	0.209 (0.185, 0.236)*	−0.209 (−0.236, −0.185)*	4.785 (4.242, 5.397)*
**Girls**				
Normal weight	−0.066 (−0.076, −0.058)	0.066 (0.058, 0.076)	0	15.046 (13.193, 17.159)
Overweight	0.379 (0.331, 0.434)	−0.674 (−0.749, −0.607)	0.295 (0.251, 0.347)	1.483 (1.335, 1.647)
Obesity	0	0.290 (0.241, 0.349)	−0.290 (−0.349, −0.241)	3.450 (2.869, 4.149)
**Boys**				
Normal weight	−0.111 (−0.124, −0.099)	0.111 (0.099, 0.124)	0	9.016 (8.052, 10.095)
Overweight	0.317 (0.280, 0.358)	−0.551 (−0.605, −0.502)	0.235 (0.204, 0.270)	1.814 (1.652, 1.991)
Obesity	0	0.172 (0.146, 0.201)	−0.172 (−0.201, −0.146)	5.832 (4.972, 6.840)

^*^The difference between boys and girls were significant (p < 0.05). Results were estimated by the null model (with no covariates included).

With the separate addition of each covariate into the null model, the goodness-of-fit consistently improved, as indicated by smaller AIC and *p* < 0.05 (Supplementary Table 1).

**Figure 1:**
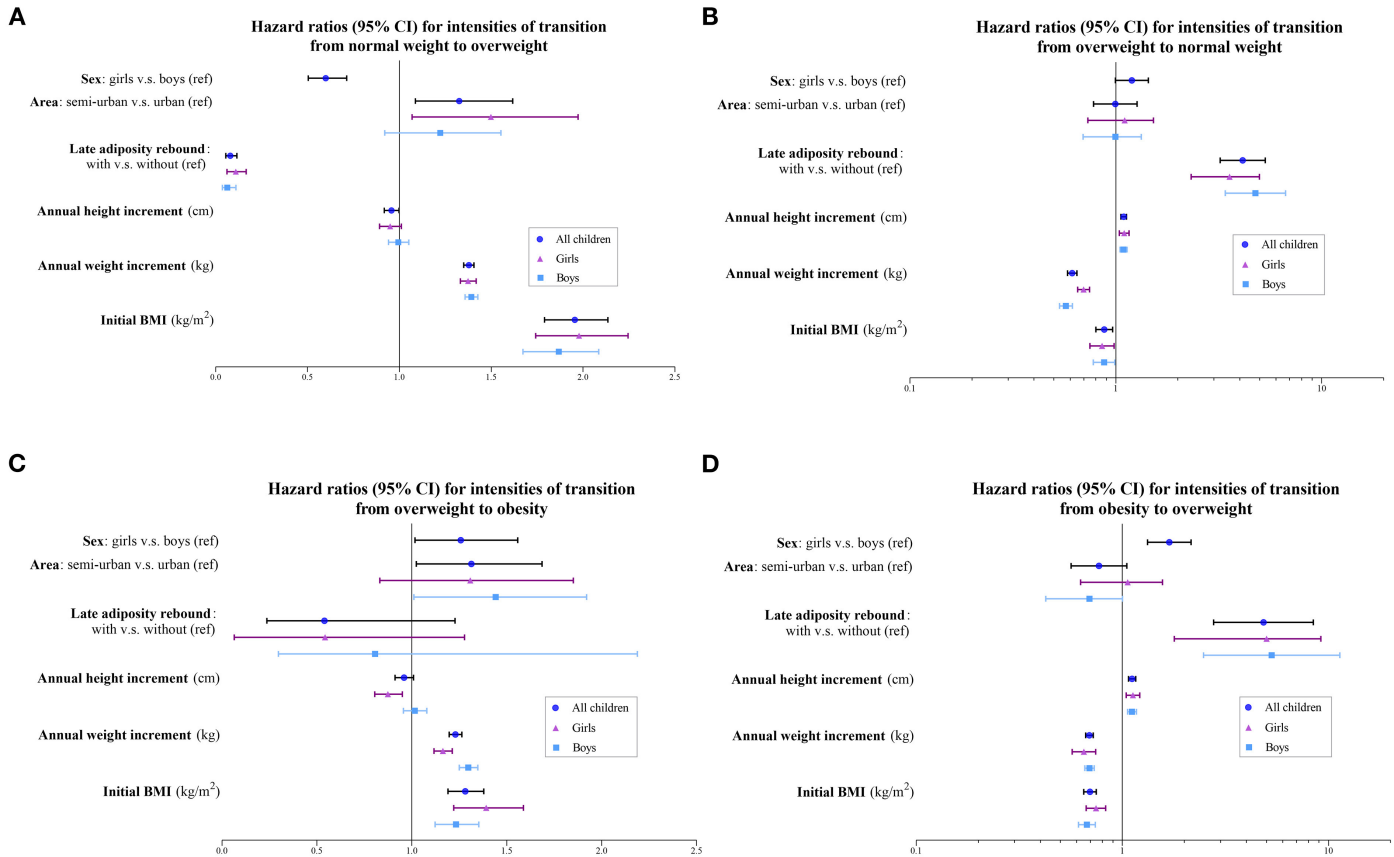
Hazard ratios and 95% confidence intervals for intensities of transition **(A)** from normal weight to overweight; **(B)** from overweight to normal weight; **(C)** from overweight to obesity; and **(D)** from obesity to overweight. BMI, body mass index.

Figure 1 shows the effects of covariates on rates of transition. Schools in semi-urban areas, higher AWI, and higher initial BMI were significant risk factors for transitioning from a lower to higher weight status. Male sex, not having late AR, and lower AHI were associated with significant increases in the rate of transitioning from normal weight to overweight. In contrast, for reversal from a higher weight status, not having late AR, lower AHI, higher AWI, and higher initial BMI were associated with lower transition rates. Additionally, the transition rate from obesity to overweight was lower in boys.

According to the null model, at age 11 years, the estimated prevalence of normal weight, overweight, and obesity was 68.2, 15.4, and 16.4%, respectively, for all children (59.5, 18.7, and 21.8% for boys; 76.9, 12.1, and 11.0% for girls, respectively). Compared with observed values, the estimated prevalence of overweight was slightly lower for boys (21.2% [95% CI: 18.9, 23.5] for observed) whereas all other predicted values fell within the 95% CIs of the observed values.

## Discussion

The current study was conducted in Minhang district, Shanghai. Shanghai is an important economic center in China and the Minhang District is the second-largest in Shanghai, covering an area of 370.75 km^2^ and with a population of 2,533,300, of which more than half (50.67%) are the floating population. Thus, the area and economic development of Minhang District are comparable to those of other medium-sized cities in China (26), improving the representativeness of this study.

In this study, we used a three-state Markov model and found that, in Chinese school-age children, overweight status tended to be unstable whereas normal weight and obese status tended to persist. This conclusion is supported by studies from Portugal and the United States (Texas and Colorado) (13, 25, 27) but is inconsistent with the findings of research in Finland and Pennsylvania (United States). Both overweight and obese status were strongly maintained (1-year probability of maintenance: 76.3% for overweight and 67.7% for obesity) in the Finnish study (14), whereas school-age children categorized as overweight or obese status tended to transition to another status in Pennsylvania (12). These inconsistencies may be explained by differences in social perceptions, geography, and causes of obesity in different countries. Additionally, our transition intensity matrix suggests that, compared with boys, weight status is more unstable in girls who are obese or overweight and more stable in girls of normal weight. Overall, the critical period for weight interventions among Chinese school-age children should be at the overweight stage rather than obesity because weight loss is difficult to achieve once a child has become obese. Differently phased intervention programs can be designed specifically for boys and girls.

In this study, we also identified factors that influence the transition pattern of weight status. Our findings suggest that the prevention of unhealthy weight should start before school age and should target boys in semi-urban areas with early AR, lower AHI, higher AWI, and higher initial BMI.

First, boys had a higher rate of transitioning from normal weight to overweight than girls and were less likely to revert from obesity to overweight, which is consistent with findings from the United States and Portugal (25, 27). The critical scrutiny of girls' body shapes may play a role in this sex discrepancy; additionally, obesity in boys of school age is traditionally considered more favorable (28, 29). Furthermore, a nationwide survey found that, compared with girls, boys have more unhealthy behaviors that contribute to obesity including poor dietary habits, sedentary lifestyles, less physical activity, and longer TV viewing times (30).

Second, in our study, children in semi-urban areas were more likely to transition to a higher weight status, in line with findings from the United States (27). A Chinese national survey in 2018 also showed that the combined prevalence of overweight and obesity was higher among children and adolescents in rural areas (16.5%) than among those in urban areas (15.1%) (31), possibly owing to the lower socioeconomic status and level of parental education (32).

Third, we found that a higher initial BMI when a child starts elementary school (6 years of age) is associated with a higher rate of transitioning to an unhealthy weight status and a lower rate of reversal, implying that the weight status of a preschool child greatly influences the probability of being overweight and obese in later life. A national longitudinal study in the United States also demonstrated that more than half of obese 14-year-old children had been overweight and ~75% had been above the 70th percentile of BMI at age 5 years (17). Thus, promoting a balanced diet and maintaining a healthy weight in early childhood may help to prevent unhealthy weight status at older school ages and in later life.

Fourth, AWI significantly modified the rates of all transitions whereas AHI was only associated with a higher rate of transitioning from high to lower weight status. This finding suggests that AWI is a valuable marker of obesity during school age, even when there has been a relatively large height increment in the same year. Thus, an appropriate rate of weight gain while growing up is important.

Lastly, children with late AR more frequently reverted from overweight to normal weight and less frequently transitioned to higher weight status. This finding is in agreement with a study investigating the relationship between late AR and the progression or reversal of childhood obesity (33). Other studies have also found that children with early AR grow faster, develop earlier, and reach puberty earlier (34). Furthermore, those with early AR are estimated to have a higher BMI and risk of metabolic syndrome in the future (35, 36), consistent with the fact that AR reflects an accumulation of adipose tissue rather than lean body mass in children (35, 36). Thus, the rate and duration of BMI development in early childhood (pre-school age) are important, and the age of AR is an indicator of susceptibility to obesity.

The findings of the current study have the following implications. First, the findings of this study indicate that overweight status tends to be unstable whereas obesity is more stable, facilitating the identification of target groups for intervention. Next, the mean sojourn time can serve as a reference for analyzing the effects of intervention strategies. Furthermore, assuming that the rate of transition between weight statuses is constant, we can predict the future prevalence of each status after including additional covariates (for instance, genetic factors) (13). Moreover, this study can facilitate health economic evaluation of obesity and associated chronic diseases, by taking into account the total length of time spent at each weight status, in addition to the prevalence and mortality. For example, a longitudinal study in Colorado (United States) among 65,672 children aged 3–15 years found that children who are overweight at age 3 years can be expected to have normal weight for 4.5 years, overweight for 5.1 years, and obesity for 3.4 years over the following 13 years (13). However, in our study, we estimated that 6-year-old children would have normal weight for 4.327 years, overweight for 0.469 years, and obesity for 0.184 years during the following 5 years. Thus, the burden of childhood overweight and obesity is lower in China than that in developed countries such as the United States.

To the best of our knowledge, this was the first large longitudinal cohort study to investigate Chinese childhood weight status using a multistate Markov model. Although a previous study with a small cohort of 928 participants covering 2004–2009 also used this model (15), the epidemic of childhood overweight and obesity has since increased sharply (11).

Our study has some limitations. First, owing to limitations of the data source, we did not take into account the influence of socioeconomic status and behavioral factors such as physical activity and dietary patterns, or socio-demographic factors including nutrition and ethnicity (37, 38); this limitation can be improved upon in future studies. Second, we used criteria recommended by the World Health Organization and Group of China Obesity Task Force to categorize children's weight status at the ages of 6 years and 7–11 years, respectively, which may result in heterogeneities between baseline and follow-up. Finally, because the multistate model requires complete data, we excluded all children with missing data from the analysis. This may cause selection bias and limit the generalizability of our findings.

## Conclusion

Our study results suggest that, among Chinese children of school age, overweight status is highly unstable whereas obesity and normal weight status are more stable. Providing weight interventions for children who are overweight at the pre-school stage may yield better effects, particularly targeting boys of pre-school age in semi-urban areas who have early AR, lower AHI, higher AWI, and higher BMI. Future studies should include more potentially relevant variables to enable the exploration of crucial modifiers of the progression and improvement in weight status.

## Data availability statement

The original contributions presented in the study are included in the article/Supplementary files, further inquiries can be directed to the corresponding author/s.

## Ethics statement

The studies involving human participants were reviewed and approved by Ethics Committee of the School of Public Health and Nursing Affiliated with Shanghai Jiao Tong University School of Medicine. Written informed consent from the participants' legal guardian/next of kin was not required to participate in this study in accordance with the national legislation and the institutional requirements.

## Author contributions

XH performed the analyses, interpreted the data, and developed the manuscript. LT conceptualized this study and validated data analyses. ZW supervised this study and reviewed the manuscript. JZ designed the study, acquired the data, reviewed, and edited the manuscript. All authors have made a significant contribution to this study.

## Funding

This study was supported in part by the National Natural Science Foundation of China (Grant No. 71303156) and the Shanghai Public Health Outstanding Young Personnel Training Program (GWV-10.2-XD07).

## Conflict of interest

The authors declare that the research was conducted in the absence of any commercial or financial relationships that could be construed as a potential conflict of interest.

## Publisher's note

All claims expressed in this article are solely those of the authors and do not necessarily represent those of their affiliated organizations, or those of the publisher, the editors and the reviewers. Any product that may be evaluated in this article, or claim that may be made by its manufacturer, is not guaranteed or endorsed by the publisher.
