# Supplementary material for: Transition patterns of weight status: A cohort study of Chinese school-age children
Source: Front Public Health. 2022 Nov 7;10:942307. doi: 10.3389/fpubh.2022.942307 (PMC9677102; doi:10.3389/fpubh.2022.942307)
Supplement: Supplementary file 1 [file Table_1.docx]

Supplementary Material

**Supplementary Table 1.** AIC values of the multistate intensity models.

|  | **Total** | | **Girls** | | **Boys** | |
| --- | --- | --- | --- | --- | --- | --- |
| **Model** | *p* | AIC | *p* | AIC | *p* | AIC |
| **Total** |  |  |  |  |  |  |
| Null model |  | 9661.135 |  | 4202.507 |  | 5404.152 |
| + Sex | <0.001 | 9606.659 |  |  |  |  |
| + Area | <0.001 | 9651.946 | <0.001 | 4203.784 | <0.001 | 5400.140 |
| + AHI | <0.001 | 9604.377 | <0.001 | 4169.695 | <0.001 | 5382.101 |
| + AWI | <0.001 | 7494.119 | <0.001 | 3385.601 | <0.001 | 4020.491 |
| + Initial BMI | <0.001 | 8914.813 | <0.001 | 3839.124 | <0.001 | 5044.734 |
| + Late adiposity rebound | <0.001 | 8923.435 | <0.001 | 3916.740 | <0.001 | 4962.476 |

Note: *p* values were calculated by the likelihood ratio test comparing nested models with the null model. AIC, akaike information criterion; AHI, annual height increment; AWI, annual weight increment; BMI, body mass index.
